# Supplementary material for: Obesity dysregulates the pulmonary antiviral immune response
Source: Nat Commun. 2023 Oct 19;14:6607. doi: 10.1038/s41467-023-42432-x (PMC10587167; doi:10.1038/s41467-023-42432-x)
Supplement: Supplementary file 1 — Supplementary Information [file 41467_2023_42432_MOESM1_ESM.pdf]

Supplementary Tables and Figures

Surface markers	Conjugated monoclonal antibody	Clone	Manufacturer	Dilution
CD45^{high}	BV711	30-F11	eBioscience	1:200
CD11b^{high}	PerCP	M1170	eBioscience	1:300
CD11c-	APC	HL-3	BD Pharmigen	1:100
F480-	PE	BM8	eBioscience	1:100
Ly6G^{high}	FITC	1A8	BD Pharmigen	1:100
CD63^{high}	PEDzl	NVG-2	Biolegend	1:100
CD64 ^{high}	PECy7	X54-5/7.1	Biolegend	1:50

Supplementary Table 1: Details of surface markers and antibodies used in flow cytometry analysis.

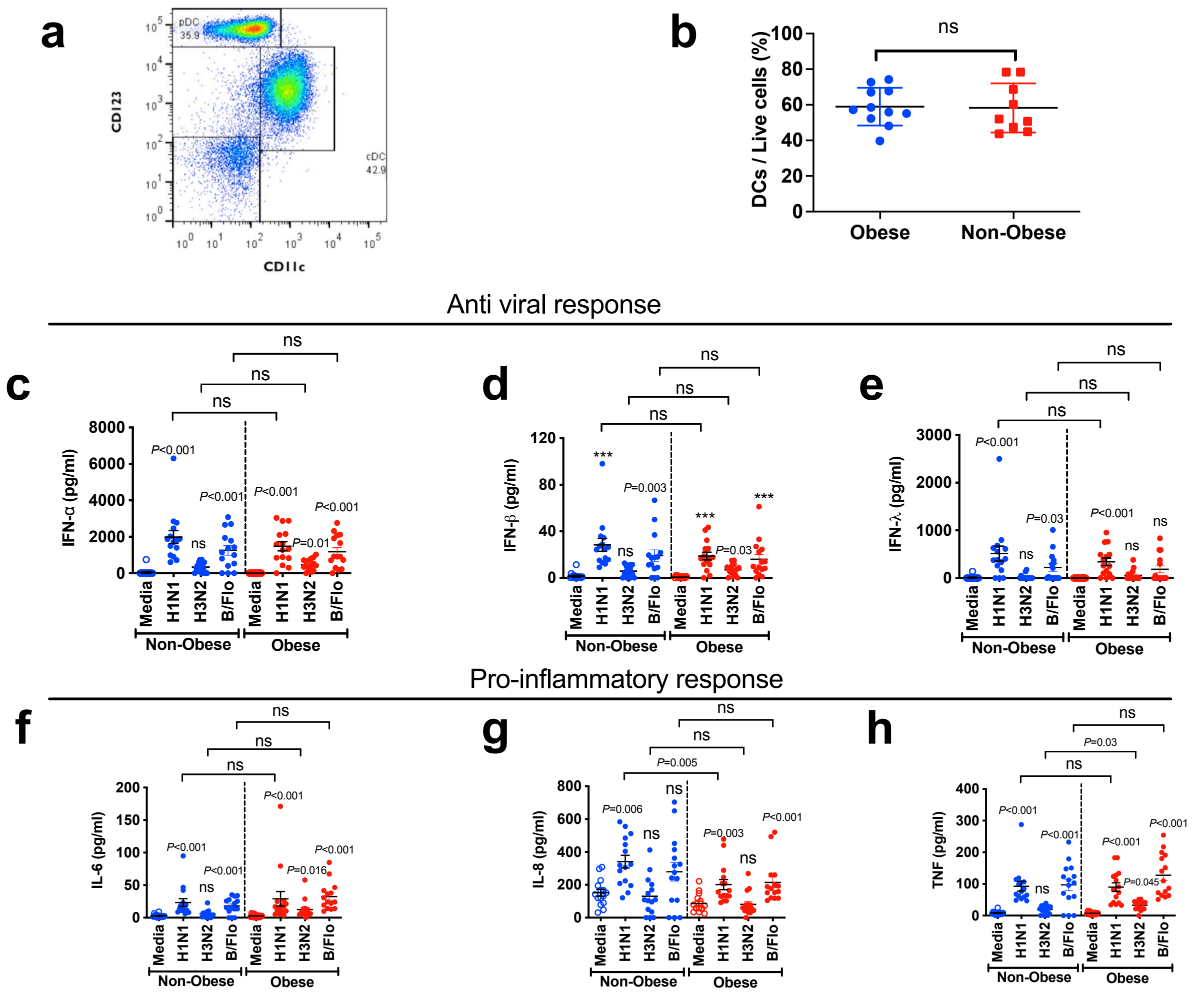

Supplementary Figure 1: Obesity does not alter dendritic cell antiviral or pro-inflammatory responses to influenza infection. Dendritic cells were isolated from 10 obese and 13 non-obese control subjects by flow cytometry (A&B), cultured and infected *ex vivo* with H1N1/09 (H1N1), seasonal H3N2 and B/Florida (B/Flo) influenza viruses. Cell supernatants were collected at 24 hours. (C) Interferon (IFN)-α, (D) IFN-β, (E) IFN-λ (F) IL-6, (G) CXCL-8/IL-8 and (H) TNF protein concentrations were quantified by multiplex ELISA. Graphs show datapoints for individual subjects with lines indicating median (IQR). Significance indicated above each group compares virus infected cells to medium-treated control cells. Brackets show comparisons between non-obse and obese subject groups. Data analysed by Kruskal Wallis with Dunn's post-test. ns= non-significant. Source data are provided as a Source Data file

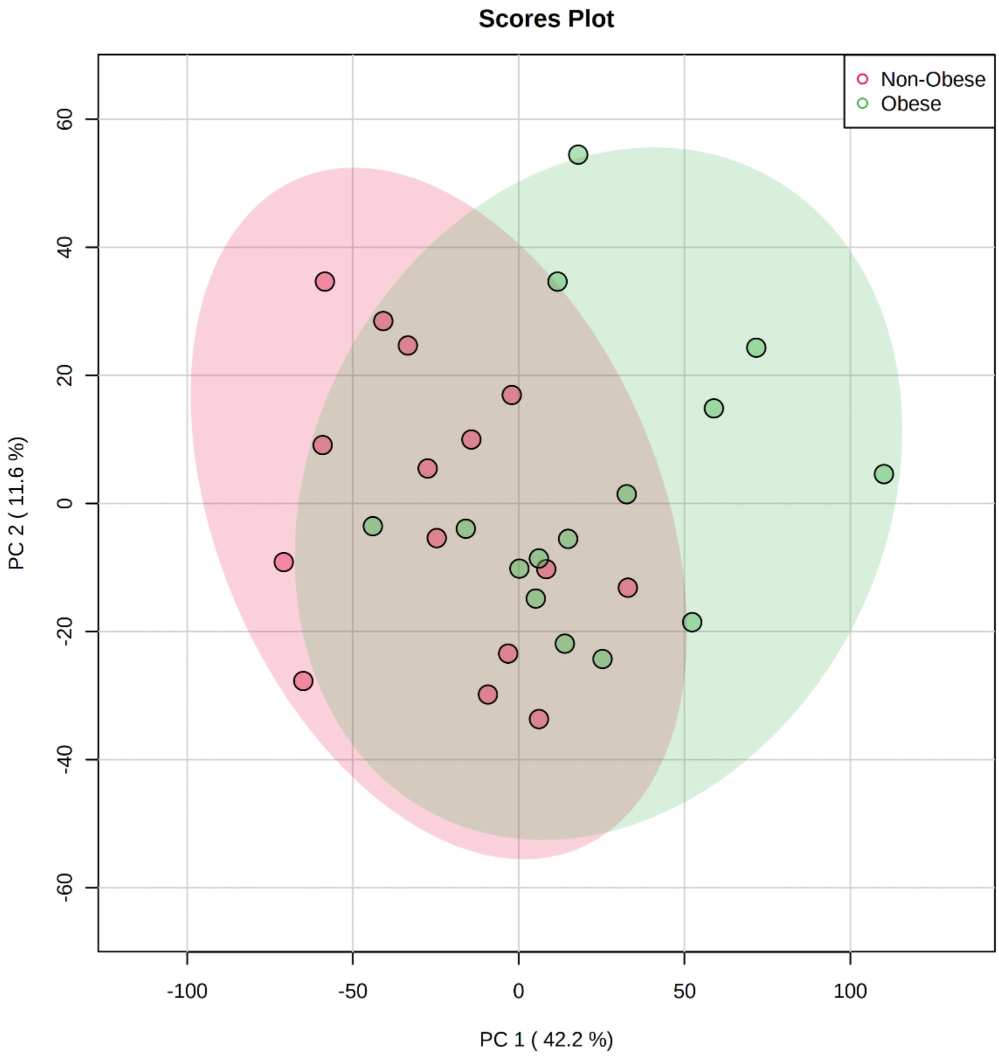

Supplementary Figure 2: Unsupervised principal components analysis scores plot of obese vs non-obese patients based on metabolite abundances.

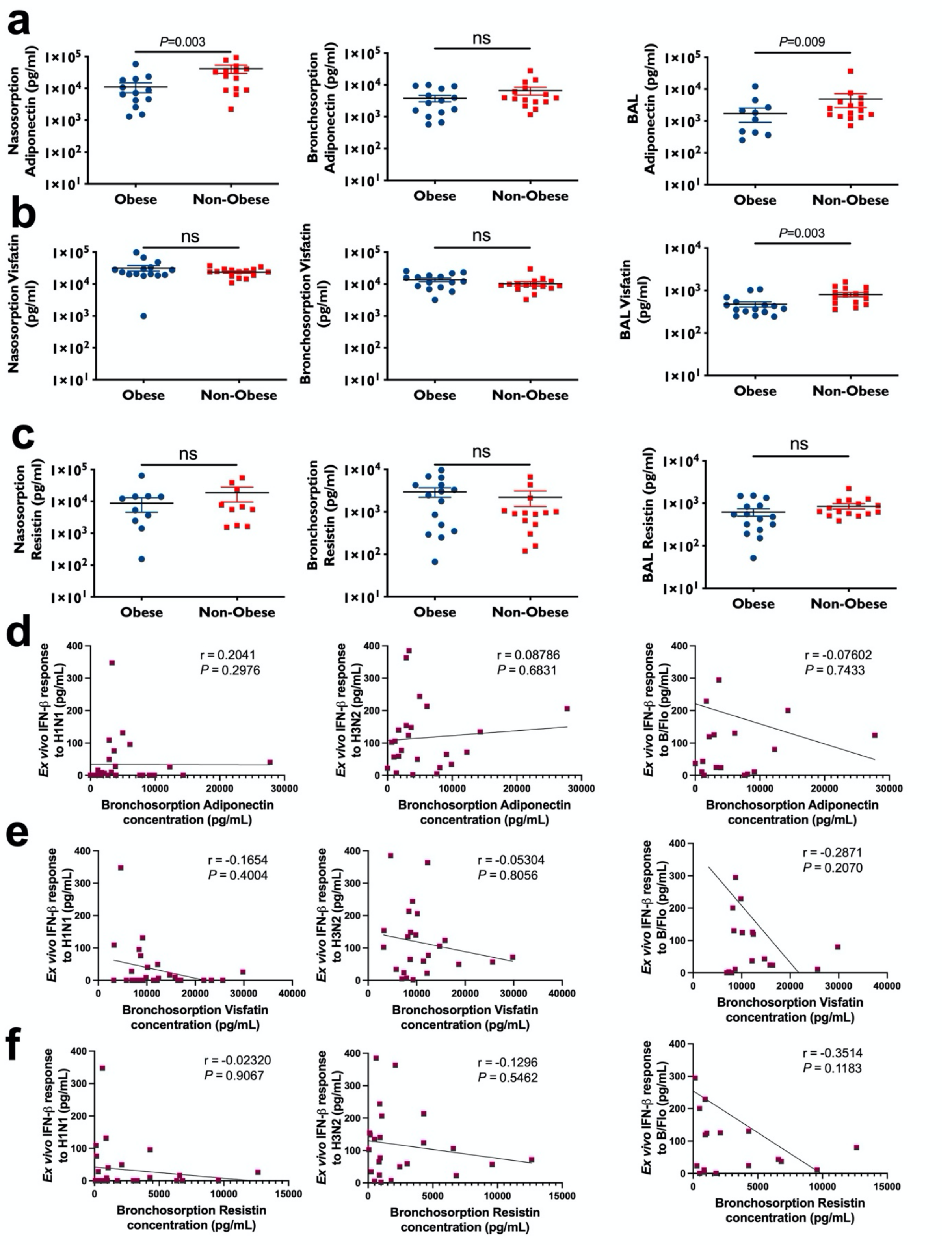

Supplementary Figure 3: Airway concentrations of adipokines and correlations with ex vivo macrophage antiviral immune responses: Concentrations of (A) Adiponectin (B) Visfatin (C) Resistin in nasosorption, bronchosorption and BAL measured by Luminex immunoassays. Correlations of (D) Adiponectin (E) Visfatin (F) Resistin with ex vivo IFN-β release by macrophages in response to H1N1, H3N2 and B/Florida influenza. Data in A-C analysed by Mann Whitney U test. Data in D-F analysed by Spearman's rank correlation test. ns = non-significant. Source data are provided as a Source Data file

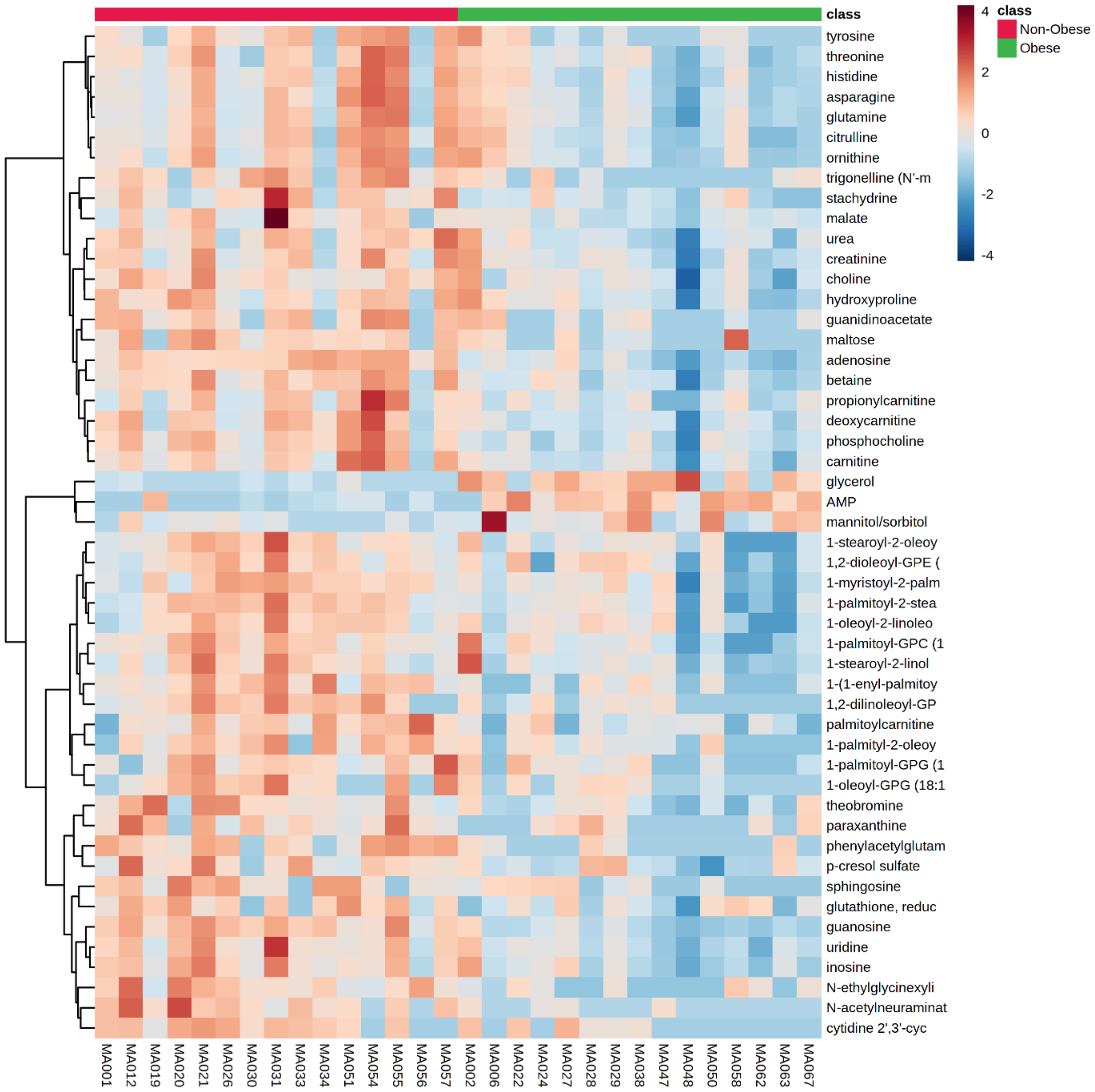

Supplementary Figure 4: An enlarged heat map (Figure 4D) for obese (green) and non-obese (red) subjects to show the top 50 metabolite correlations.

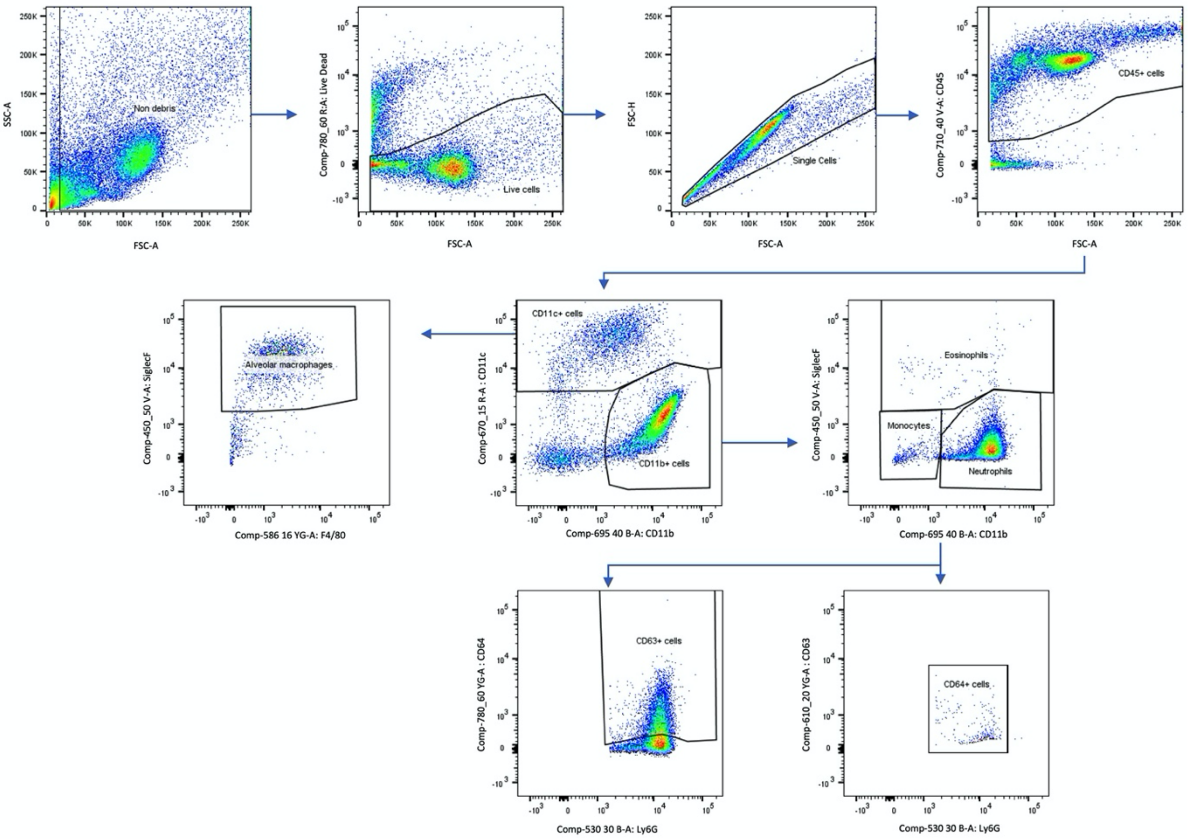

Supplementary figure 5: Flow cytometry Gating strategy for identification of neutrophils in mouse samples
