# Supplementary material for: Obesity dysregulates the pulmonary antiviral immune response
Source: Nat Commun. 2023 Oct 19;14:6607. doi: 10.1038/s41467-023-42432-x (PMC10587167; doi:10.1038/s41467-023-42432-x)
Supplement: Supplementary file 2 — Reporting Summary [file 41467_2023_42432_MOESM2_ESM.pdf]

nature portfolio

Corresponding author(s): Aran Singanayagam

Last updated by author(s): Sep 25, 2023

Reporting Summary

Nature Portfolio wishes to improve the reproducibility of the work that we publish. This form provides structure for consistency and transparency in reporting. For further information on Nature Portfolio policies, see our Editorial Policies and the Editorial Policy Checklist.

Statistics

For all statistical analyses, confirm that the following items are present in the figure legend, table legend, main text, or Methods section.

n/a	Confirmed	
☐	☒	The exact sample size (*n*) for each experimental group/condition, given as a discrete number and unit of measurement
☐	☒	A statement on whether measurements were taken from distinct samples or whether the same sample was measured repeatedly
☐	☒	The statistical test(s) used AND whether they are one- or two-sided<br>*Only common tests should be described solely by name; describe more complex techniques in the Methods section.*
☒	☐	A description of all covariates tested
☐	☒	A description of any assumptions or corrections, such as tests of normality and adjustment for multiple comparisons
☐	☒	A full description of the statistical parameters including central tendency (e.g. means) or other basic estimates (e.g. regression coefficient) AND variation (e.g. standard deviation) or associated estimates of uncertainty (e.g. confidence intervals)
☐	☒	For null hypothesis testing, the test statistic (e.g. *F*, *t*, *r*) with confidence intervals, effect sizes, degrees of freedom and *P* value noted<br>*Give P values as exact values whenever suitable.*
☒	☐	For Bayesian analysis, information on the choice of priors and Markov chain Monte Carlo settings
☒	☐	For hierarchical and complex designs, identification of the appropriate level for tests and full reporting of outcomes
☒	☐	Estimates of effect sizes (e.g. Cohen's *d*, Pearson's *r*), indicating how they were calculated

Our web collection on statistics for biologists contains articles on many of the points above.

Software and code

Policy information about availability of computer code

Data collection	*Provide a description of all commercial, open source and custom code used to collect the data in this study, specifying the version used OR state that no software was used.*
Data analysis	Metabolomics data processing steps were completed using the online Metaboanalyst 5.0 platform (https://www.metaboanalyst.ca/).<br><br>All other statistical analysis was performed using GraphPad Prism version 9.

For manuscripts utilizing custom algorithms or software that are central to the research but not yet described in published literature, software must be made available to editors and reviewers. We strongly encourage code deposition in a community repository (e.g. GitHub). See the Nature Portfolio guidelines for submitting code & software for further information.

Data

Policy information about availability of data

All manuscripts must include a data availability statement. This statement should provide the following information, where applicable:

- Accession codes, unique identifiers, or web links for publicly available datasets
- A description of any restrictions on data availability
- For clinical datasets or third party data, please ensure that the statement adheres to our policy

The metabolomics data generated in this study have been deposited in a database available at https://doi.org/10.17028/rd.lboro.23939772.

Research involving human participants, their data, or biological material

Policy information about studies with human participants or human data. See also policy information about sex, gender (identity/presentation), and sexual orientation and race, ethnicity and racism.

Reporting on sex and gender	Data from human participants in the bronchoscopy study derives from male and female sex individuals. There was no significant difference between our experimental (obese) and control (non-obese) groups in terms of % males and females. These data are reported in Table 1.<br><br>The MOSAIC study recruited unselected subjects admitted to hospital with influenza infection. There was a non-significant trend towards greater % females in the obese subgroup. These data are reported in Table 1.
Reporting on race, ethnicity, or other socially relevant groupings	Human participants in the brocnhoscopy study included a mix of caucasian, asian and black individuals. These data are reported in Table 1.<br><br>The MOSAIC study also included a mix of caucasian, asian and black individuals and data are reported in Table 2.
Population characteristics	Data about comorbidities and therapies are reported in Table 1 (bronchoscopy study) and Table 2 (MOSAIC study)
Recruitment	For the bronchoscopy study, individuals were prospectively recruited from the bariatric surgery service at Imperial College Healthcare NHS Trust as part of a case-control study (bronchoscopy study). Cases were recruited at bariatric surgery clinics and bariatric surgery education seminars held at Imperial College NHS Trust. Controls were recruited via advertisements in local newspapers and posters around the Imperial College Campus. Potential controls that contacted the study research nurse via telephone or e-mail were then invited to the Imperial College Respiratory Research Unit (ICRRU) for an initial screening visit to evaluate eligibility.<br><br>In the MOSAIC study, unselected adult patients presenting with influenza-like symptoms were recruited between December 2009 and March 2011 from three hospitals in Liverpool and the Wirral (north-west England) and six hospitals in London (MOSAIC study).
Ethics oversight	Human bronchoscopy study: The study received ethical approval from the Surrey Borders Research Ethics Committee (approval number 12/LO/1812). Informed consent was obtained from all participants.<br><br>The Mechanisms of Severe Acute Influenza Consortium (MOSAIC) study of hospitalised influenza infections: The study was approved by the NHS National Research Ethics Service, Outer West London REC (09/H0709/52, 09/MRE00/67). Informed consent was obtained from all participants.

Note that full information on the approval of the study protocol must also be provided in the manuscript.

Field-specific reporting

Please select the one below that is the best fit for your research. If you are not sure, read the appropriate sections before making your selection.

☒ Life sciences ☐ Behavioural & social sciences ☐ Ecological, evolutionary & environmental sciences

For a reference copy of the document with all sections, see nature.com/documents/nr-reporting-summary-flat.pdf

Life sciences study design

All studies must disclose on these points even when the disclosure is negative.

Sample size	We were unable to perform a power calculation as this is the first study of its kind; however, previous publications have demonstrated significant intergroup differences in interferon (IFN) production with numbers fewer than 15 per study arm. For example, Contoli et al (PMID: 16906156) demonstrated an asthma-associated type III IFN deficiency in BAL cells and hBECs using nine cases and nine controls. Additionally, Mallia et al demonstrated an IFN deficiency in COPD using 13 subjects and 13 controls (PMID: 20889904). Given the resources available for the study, 15 study subjects per arm was also a practical and achievable number.
Data exclusions	Nil
Replication	Independent replication in a validation human cohort was not feasible for this study.
Randomization	Experimental groups were defined by presence or absence of obesity and therefore randomisation not possible/relevant
Blinding	All experimental assays were performed with the researchers blind to the treatment received

Reporting for specific materials, systems and methods

We require information from authors about some types of materials, experimental systems and methods used in many studies. Here, indicate whether each material, system or method listed is relevant to your study. If you are not sure if a list item applies to your research, read the appropriate section before selecting a response.

Materials & experimental systems

n/a	Involved in the study
☐	☒ Antibodies
☒	☐ Eukaryotic cell lines
☒	☐ Palaeontology and archaeology
☐	☒ Animals and other organisms
☐	☒ Clinical data
☒	☐ Dual use research of concern
☒	☐ Plants

Methods

n/a	Involved in the study
☒	☐ ChIP-seq
☐	☒ Flow cytometry
☒	☐ MRI-based neuroimaging

Antibodies

Antibodies used	CD45: BV711 clone 30-F11 eBioscience (dilution 1:200)<br>CD11b PerCP clone M1170 eBioscience (dilution 1:300)<br>CD11c APC clone HL-3 BD Pharmigen (dilution 1: 100)<br>F480 PE Clone BM8 eBioscience (dilution 1:100)<br>Ly6G FITC clone 1A8 BD Pharmigen (dilution 1:100)<br>CD63 PEDzl clone NVG-2 Biolegend (dilution 1:100)<br>CD64 PECy7 X54-5/7.1 Biolegend (dilution 1:50)
Validation	Please see manufacturer's websites for relevant details

Animals and other research organisms

Policy information about studies involving animals; ARRIVE guidelines recommended for reporting animal research, and Sex and Gender in Research

Laboratory animals	Female mice (6–8 weeks of age) on a BALB/c background house in individually ventilated cages within specific pathogen free conditions. Mice had access to food and water ad libitum with 12 hour alternating light/dark cycles at temperature of 20-24 degrees celsius
Wild animals	No wild animals were used in this study,
Reporting on sex	Female mice alone were used in our animal experiments. This is in line with our prior work in these models which have typically used females for consistency. Male mice have an increased propensity to fight which can invoke a stress response which may affect data generated.
Field-collected samples	No field samples were collected in this study.
Ethics oversight	All animal work was performed under the authority of the UK Home Office outlined in the Animals (Scientific Procedures) Act 1986 after ethical review by Imperial College London Animal Welfare and Ethical Review Body (project licence PP4051423).

Note that full information on the approval of the study protocol must also be provided in the manuscript.

Clinical data

Policy information about clinical studies

All manuscripts should comply with the ICMJE guidelines for publication of clinical research and a completed CONSORT checklist must be included with all submissions.

Clinical trial registration	n/a - not a clinical trial
Study protocol	n/a - not a clinical trial
Data collection	Clinical rescruitment and data collection occurred from 13th June 2013 to 6th January 2015
Outcomes	Our primary outcome was the ex vivo antiviral immune response to influenza measured in macrophages, bronchial epithelial cells and plasmacytoid dendritic cells

Flow Cytometry

Plots

Confirm that:

☒ The axis labels state the marker and fluorochrome used (e.g. CD4-FITC).

☒ The axis scales are clearly visible. Include numbers along axes only for bottom left plot of group (a 'group' is an analysis of identical markers).

☒ All plots are contour plots with outliers or pseudocolor plots.

☒ A numerical value for number of cells or percentage (with statistics) is provided.

Methodology

Sample preparation	BAL cells were stained with the Live/Dead Fixable Near-IR-Dead Cell staining kit (Invitrogen) for 20 minutes in PBS prior to blockade with anti-CD16/CD32 Fc receptor block (BD Pharminogen) for 20 minutes. Cells were then washed in PBS containing 0.1% sodium azide and 1% BSA followed by staining for surface markers at 40C for 30 minutes. Cells were subsequently washed in PBS containing 0.1% sodium azide and 1% BSA before fixation in 2% paraformaldehyde
Instrument	BD LSRFortessa
Software	Collection of Data: BD FACSDiva<br>Analysis of Data: FlowJo
Cell population abundance	n/a - no sorting
Gating strategy	Shown in Supplementary Figure 5

☒ Tick this box to confirm that a figure exemplifying the gating strategy is provided in the Supplementary Information.
